# The Present and Future of Graduate Medical Study

**Published:** 1910-04-09

**Authors:** Robert Kutner

**Affiliations:** Director Kaiserin Friedrich Haus, Berlin, General Secretary International Committee for Medical Extension Courses.


### SPECIAL ARTICLES.

### THE PRESENT AND FUTURE OF GRADUATE MEDICAL STUDY.

By PROFESSOR ROBERT KUTNER, M.D., C.V.O., Director Kaiserin Friedrich Haus, Berlin, General Secretary International Committee for Medical Extension Courses.

At the outset it may be stated that no single individual or nation can lay claim to the merit of having initiated post-graduate medical instruction. No practitioner, no country, can claim to have created post-graduate medical education as it exists to-day; it has resulted from the efforts of a number of men who have for many years worked in unselfish devotion for the interests of their colleagues. If we could conceive the gigantic idea of all civilised nations combining to erect an edifice reaching to the heavens, we should at once understand that the various countries would supply vastly heterogeneous building material according to available natural products and local conditions. It is precisely similar with post-graduate medical education. According to the peculiarities of the various countries, their historical and cultural development, the age of their universities, and, more particularly, the nature of existing organised medical education, the character of the present post-graduate education has assumed a different shape in the various countries where this branch of learning has at all been cultivated. It would be idle, therefore, to discuss questions of priority—who were the first pioneers or the most effective workers. At this Peace Festival of Science * it behoves us to recognise without envy all good results, wherever they may have originated, and cheerfully to combine in order to bring our former achievements to as high a level of perfection as may lie within us.

### The Present State of Post-Graduate Study.

Permit me to begin with a short description of what has up to the present been accomplished in organising post-graduate education, what the future objects of post-graduate education should be, and, finally, to submit a scheme for the closer co-operation of all forces conducive to the attainment of our common objects.

To commence with Germany there were in that country up to the year 1900, only the so-called vacation classes devoted to post-graduate instruction. These classes were conducted by members of the faculties of several universities in the spring or autumn, lasting three to four weeks, and were frequented not only by German practitioners but even more so by those coming from foreign countries. All these organisations were modelled after the *Berlin Dozenten-Vereinigung für Ferienkurse*, which has thereby acquired the lasting merit of having furthered our common interests. The fact, however, that these vacation classes were restricted to short periods, compelling the visitors to devote their whole time to the classes and clinical lectures, prevented many practitioners, even local residents, from attending. The difficulties were even greater for those practitioners who had to undertake more or less prolonged journeys to attend these courses, and in the meantime to give up their practice during their absence from home. Considering the sacrifice in time, the loss of practice, the travelling and living expenses, and finally the fees charged by the instructors, it is evident that many practitioners were absolutely debarred from the benefits of such post-graduate education. Then an ideal suggested itself, for the realisation of which the late Empress Frederick had greatly exerted herself—to give all practitioners the same opportunity for post-graduate education as existed in a small degree for those holding Government appointments and for military surgeons. To this end it was necessary to establish a systematic organisation of clinical classes and lectures, which would enable every German practitioner to remain in touch with the progress in medicine without being compelled to make considerable financial and other sacrifices.

### The German Example.

Led by these considerations, a Central Committee for Medical Post-Graduate Instruction, founded on May 18, 1900, under the auspices of the Prussian Ministry of Public Instruction, resolved to create organisations for continued medical education, which should be (1) gratuitous; (2) situate at or near the places of residence of attending practitioners; (3) available at times most favourable for individual requirements. This necessitated the creation of numerous scientific centres in large cities, each of which had to be endowed with a reputation for authoritative teaching within a given district. The local members of the faculties and the pathological hospital material had to be placed at the disposal of these organisations, and from these requirements it became evident, as a matter of course, that the communal and other public hospitals, together with their directors, would have to be in the centre of the local organisations. This applied not only to non-university towns, but also to university towns, as the State institutes are primarily devoted to the instruction of students, and have therefore but scant pathological material for purposes of post-graduate instruction. An important innovation in this connection is the establishment of Academies for Practical Medicine. The first of these was created in Cologne in 1904; the second in Düsseldorf in 1907. These academies are in close contact with the Central Committee for Post-Graduate Instruction and its objects. This close relation between the Central Medical Committee and the academies assures harmonious co-operation, the academies thereby becoming important local centres of the entire movement.

It would lead me too far to describe all the various stages through which this work had to pass

* The address was delivered at the foundation of the International Committee for Medical Graduate Education at the Medical Congress, Budapest, last year, and we are largely indebted to *The Post Graduate* for the report.

in order to arrive at its present status, and I will confine myself to a few data which will demonstrate the fact that post-graduate medical education has obtained a strong foothold throughout Germany unequalled by any other country. In 48 large cities of the German Empire there are now regular annual gratuitous post-graduate and clinical lectures for practitioners. These 48 towns are situated in the following ten Federal States: Prussia, Bavaria, Saxony, Württemberg, Baden, Mecklenburg-Schwerin, Hamburg, Bremen-Oldenburg, Alsace-Lorraine, and Thüringia. In each of these Federal States there is a State Committee, which organises the instruction in the towns situate within its jurisdiction. All these State Committees form part of the "Imperial Committee for Medical Post-Graduate Instruction," which takes all questions into consideration, and decides on various subjects. The State Committees, as well as the Imperial Committee, receive appropriations from their respective governments to defray expenses. Following the example of the physicians, the German dentists have established a similar extensive organisation, which institutes gratuitous dental classes and lectures in eight large German cities.

The picture I am giving you would be incomplete without mention of two other institutions closely related to the post-graduate movement: (1) The Empress Frederick House, and (2) the Journal for Post-Graduate Medical Instruction.* The journal is the official organ of all State Committees and of the Imperial Committee, above referred to; it is further charged with the important task of making these gratuitous lectures accessible to all practitioners who, from whatever reason, are unable to attend the lectures at the appointed time. As the journal is the literary centre, so is the Empress Frederick House the structural centre for the entire German post-graduate movement. In order to satisfy all the requirements incumbent upon it, the Empress Frederick House is provided with the following arrangements:—

1. Clinical Instruction Rooms for Physicians—namely, Model rooms and laboratories for instruction in practical work in all sciences allied to modern medicine, as in clinical chemistry, microscopy, bacteriology röntgenography, and scientific photography; furthermore, a large auditorium for lectures on clinical subjects. (2) Collections of objects aiding in medical instruction, notably the State Collection of Medical Instruction Books, with the special departments for a medico-historical collection; collection of hospital instruments and furniture, and a collection of over 1,000 casts, all of which objects can be used gratuitously, and they are also loaned. (3) The Organisation Offices: rooms serving the purposes of administration for the Prussian State Committee and the Imperial Committee for Post-Graduate Instruction. (4) Office combining Medical Science and Practical Technique: A "Permanent Exposition in the interests of Medico-Technical Industry," in which physicians will find all instruments and apparatus they may have to use in surgery, electro-therapeutics, ophthalmology, etc. (5) Exposition Office: A room which can be used free of expense for scientific expositions for special purposes in medical instruction. (6) Office of Information: where free information is given on all post-graduate classes in the German Empire, on all other affairs relating to post-graduate matters, on all medical institutions, hospitals, collections, etc., in Germany, together with all particulars as to time and conditions of inspection. The House was erected with the aid of purely voluntary contributions which were all collected in 1903. The total expense amounted to nearly 1,500,000 marks. In the presence of their majesties the Emperor and the Empress, the House was formally opened on March 1, 1906. The annual cost of maintenance amounts to about 50,000 marks and is defrayed (1) by the State Government, which has rented a part of the House for university purposes; (2) by the rentals charged to exhibitors in the Permanent Exposition; (3) by the interest on the original capital which was not utilised for building purposes.†

* *Zeitschrift für Aerztliche Fortbildung.*

### America.

In America medical post-graduate instruction has found a firm foothold in the Post-Graduate Medical Schools. The most prominent is the New York Post-Graduate Medical School, a model institution for the entire United States, and indeed for all similar institutions of the rest of the civilised world. It should not be forgotten, however, that post-graduate education in America has developed under different conditions from those in Germany. As is well known, there was a time when the clinical education of American practitioners, especially that of the specialists, left much to be desired. This drawback was enhanced by the fact that practically anybody was allowed to practice medicine, and it was with a view to eradicating this evil, and especially to impart a more profound knowledge of the specialties, that the post-graduate medical schools were founded.

The first and still the most prominent is the New York Post-Graduate Medical School,‡ founded by D. B. St. J. Roosa, April 4, 1882. Hardly had the idea of medical post-graduate institutions taken a firm footing in the shape of this school, than other similar schools followed in its wake. New York produced the New York Policlinic, which thrives well upon the model of the New York Post-Graduate and has become an important institution. Other schools were established in Chicago and Philadelphia, and soon there were more than a dozen of them throughout the country. During the last ten years many small similar schools were added, some of which, however, were equipped with insufficient facilities and instructors to conduct them on a large scale; their classes, in fact, may be looked upon as a kind of permanent summer course. Systematic post-graduate instruction now exists in the following institutions (the figures in brackets indicating the year of their foundation):

† A full description of the Institution was given in a series of articles in The Hospital, 1909, pp. 369, 421, 547, 571.

‡ A description of this school appeared in The Hospital of April 24, 1909, p. 113, and May 8, 1909, p. 165.

(1) New York Post-Graduate Medical School and Hospital (1882), 218 beds, about 572 matriculates annually.

(2) New York Policlinic, 105 beds, 300 matriculates.

(3) Post-Graduate Medical School and Hospital of Chicago (1899), 100 beds, 300 matriculates.

(4) Chicago Policlinic (1899), 110 beds, 290 matriculates.

(5) Philadelphia Policlinic and College for Graduates, 150 beds, 170 matriculates.

(6) New Orleans Policlinic (1888), 175 matriculates.

(7) Illinois Post-Graduate Medical School (1907), 95 beds, 65 matriculates.

(8) New York School of Ophthalmology and Otology (1881), 147 beds, 47 matriculates.

(9) San Francisco Policlinic and Post-Graduate School of Medicine (1889), 30 matriculates.

(10) Chicago Eye, Ear and Throat School, 40 matriculates.

Besides there are a few other smaller institutes, and the Army Medical School which is reserved for military surgeons.

The medical faculties of some large universities, such as the Johns Hopkins, Harvard, Columbia, and Chicago Universities, have also arranged very excellent post-graduate courses for practitioners, but not in a sufficiently systematic manner to attract a large number of matriculates.

The ways and means adopted by the various post-graduate schools vary. In a general way the development of the New York Post-Graduate School is the prototype for all other schools. The fact especially that school and hospital form one whole has been recognised as the only correct method for America, as this not only allows the greatest possible concentration and consequent saving in time and expense to the matriculates, but also admits inclusion of examination of patients, laboratory investigations, and routine observations in the service of the school. This is a point on which the New York Post-Graduate School has placed from the first the greatest weight. With the exception of the leading universities, such as Johns Hopkins, Harvard, Jefferson, and others, most American colleges, even those of high scientific standing, still labour under the disadvantage that the hospitals in which the academic clinical instruction is given do not belong to the university proper. This separation of interests, both in a local and administrative way, interferes with systematic exploitation of available material. On the other hand, in the New York Post-Graduate Medical School matriculates have the advantage of studying patients in all stages of disease from the moment of their admission to the hospital up to their cure, or—as may also happen—up to death and autopsy, just as in the German university clinics.

### Germany and America: A Comparison.

The striking difference between America and Germany is however this: in America special hospital medical schools were created for purposes of initial and progressive instruction; in Germany there already existed excellent hospitals and through the medium of our organisation we equipped them with post-graduate instruction, and so it happened that we were able to create in a short time the large number of 48 teaching centres without incurring very heavy expense. Another fundamental difference is that the fees for American post-graduate classes and lectures are rather high, whereas all the classes and lectures in Germany are free of expense.

The plan upon which the instruction is conducted at the New York Post-Graduate is approximately characterised by the following facts: The faculty consists at present of 40 ordinary professors, with whom 35 adjunct professors are associated. The latter act in the absence of the ordinary professor, and their services are especially required during the summer term. Besides there are 84 instructors and about 100 clinical assistants. With the exception of the professor of pathology and his assistants, members of the faculty receive no payment for the lectures. A fee charged for the classes in the specialties go half to the institute and half to the instructor. Only graduates are admitted to the lectures, and they matriculate either for a year or for a term. The winter term lasts from October 1 to June 1. The summer term is shorter and less frequented. Practitioners who have only a limited time at their disposal may matriculate for 12 or six weeks. Such a matriculation entitles members to attend all the lectures and a number of classes for a period of six weeks to three months. Besides there are separate classes with a restricted number of members, for which a special ticket must be taken. Many matriculates attend regularly every year for a few weeks, and the arrangement is frequently made that practitioners in general practice attend in the course of time all the classes held in the most important specialties. Others again return from time to time to the same classes in order to keep in touch with the progress made in the meantime in their special branch of practice. Of great importance are the operative classes. At first matriculates are required to go through the anatomical and surgical part of their duties on the cadaver; next they assist in minor and then in larger operations; and finally they perform the operations themselves under the supervision of the instructor. Operative classes are held in general surgery, ophthalmology, otology, rhinology, and gynæcology. The special courses in bronchoscopy, œsophagoscopy, cystoscopy, catheterisation of the ureters, etc., are always well attended.

For single courses the fees vary, according to the subject and the duration of the class, between $10 and $200. Generally speaking, it may be said that the universities do comparatively very little in the way of post-graduate instruction, and the post-graduate schools predominate in this respect.

### Hungary.

The hospitable country to which we are indebted for this Convention possesses since 1883 the institution of vacation classes for post-graduate medical instruction. However, an organised post-graduate service cannot be said as yet to have been inaugurated there. It is only since the deserving general secretary of this Congress, Dr. Emil von Gross, took the matter in hand and organised the movement upon the principles of the German arrangements, that a few centres of instruction were created in Hungary (Budapest, Pressburg, and Klausenburg) under a State Committee which has its seat in Budapest. The following is a short description of the organisation in Budapest which has served as a model for those in Pressburg and Klausenburg.

The instruction in Budapest is given by professors and instructors of the University. At least two groups are formed each year with 25 matriculates each, the latter having the privilege of selecting any one of these groups, should there not be a sufficient number of matriculates for the two. The number of groups can be increased, should the number of applicants demand it. In each cycle of classes the principal subjects are taught—internal medicine, surgery, obstetrics and gynæcology, and several specialties. The classes are under the charge of an organisation committee appointed from year to year, and are gratuitous, but to cover the working expenses a subscription of 20 kronen is levied. They are well attended, not only by general practitioners but also by physicians who hold Government positions. They embrace all medical subjects, last two months, and take place once a week. The members have the privilege of reduced railway rates on the Hungarian State railways.

### Austria.

In Vienna the "Medical Chamber" in connection with the Vienna medical faculty created, in 1906, after the German model, an organisation for medical post-graduate instruction which owes much to the efforts of Dr. Thenen, of Vienna. The instructors make no charge for their services. Those of the class members who belong to the Medical Chamber must pay to the latter for each course of instruction a subscription fee of six or three kronen, according to whether the course occupies 12 or six hours; otherwise there is no charge for attendance. Practitioners not being members of the Vienna Medical Chamber are allowed to attend, provided there is room and the instructor permits. Their fee is 20 kronen for a 12-hour course and 10 kronen for a six-hour course, the money being payable direct to the instructor. No certificates are issued testifying to the attendance. The classes are very well attended, and are repeated every year.

In Graz free classes have existed for three years. Thanks to the efforts of the local practitioners and the courtesy of the faculty of the University, notably of Professors Dimmer, Von Hacker, and Praussnitz, it was possible, in the beginning of the summer of 1907, to prepare for the first time a programme for medical post-graduate classes, which was forwarded to the practitioners of the Austrian alpine countries, and beyond. To cover actual expenses, a fee of 20 kronen is levied.

### Russia.

1. I am indebted to Professor Tilding, of St. Petersburg, Director of the Imperial Clinical Institute of the Grand Duchess Helena Pawlowna, for the following communications concerning the post-graduate medical service in the above institute, over which he presides. The institute was opened May 21, 1885, with permanent departments for internal medicine, surgery, gynæcology, pathological anatomy, and bacteriology. The importance of the Institute will be understood from a few particulars concerning its faculty. At present there are seven ordinary professors, who draw the salary of a university professor (3,000 roubles), for which they have to deliver at least three lectures a week; there are also eight adjunct professors and six instructors, who have no fixed salary. There are also 20 assistants who hold private classes. Matriculates pay a fee of five to ten roubles for each course. A recent arrangement is that each matriculate pays 10 roubles per term, for which he is entitled to attend all the clinics and also the pathological laboratory.

The clinics are held throughout the entire term, the classes generally every two months. At the request of matriculates, classes have recently been held every four weeks, especially for less important subjects (operative cases in surgery, gynæcology, obstetrics, ophthalmology, clinical technique, etc.). No control is exercised over the attendance, nor are there examinations or certificates. Upon request, however, a document will be issued stating what courses were subscribed for, in order to facilitate accountancy with the local districts, but no opinion is expressed as to results attained.

2. There are two more institutes in Russia devoted to medical post-graduate instruction. One is the Imperial Clinical Obstetrical Institute in St. Petersburg. To a certain extent the classes held here supplement those held in the Institute of the Grand Duchess Helena Pawlowna. The professors attached to the Imperial Institute hold lectures and classes from the beginning of October to April, lasting two months each; physicians are also admitted to the obstetrical and gynæcological operations and assist in the clinical departments in diagnosis and therapy. The institute, which was rebuilt at the initiative of its director, Privy Councillor Dr. Ott, is a first-class model, satisfying all requirements, liberally tending to the comfort of patients, and applying the latest discoveries in technique. Most gynæcological and obstetrical classes last two months and are held from the beginning of October to the beginning of April. About 250 to 300 physicians attend the classes annually.

3. The third institution is the Gynæcological Institute for Physicians in Moscow. Under the circumspect and energetic direction of the well-known Russian gynæcologist, Professor W. Snegireff, who has acted in this capacity from the beginning, the institute enjoys the reputation of a central seat of gynæcological learning and investigation. Twice yearly medical post-graduate classes are held there in theoretical and practical gynæcology, one from February 1 to April 1, and one from October 1 to December 1. The number of matriculates is restricted, not more than 20 to 25 being admitted.

4. Finally, it may also be stated that the Council of the Russian Pirogoff Medical Society is planning the foundation of an institute for social hygiene.

### Italy.

In Italy there has been an institute for post-graduate medical education since 1905, which to a certain extent represents an academy for medical post-graduate instruction, for no other term can be applied to the hospital medical school for practitioners at Milan, which is conducted like our own academies. A practitioner who has obtained the degree of M.D. at an Italian or foreign university and desires to perfect his knowledge in any particular branch of the medical sciences may become a member of the classes. Members receive either a simple certificate stating the particulars of attendance, application and success, or a special post-graduate diploma which will be signed by the president of the faculty and countersigned by the professors of the classes attended. The certificate of attendance is issued without examination, provided the member has been a regular visitor. In order to obtain the post-graduate diploma members must submit to an examination in the subjects for which it is demanded. For the post-graduate courses the fees range from 10 lira to 30 lira, according to whether the hearer attends a single course for half a year or a full year. Some time ago I took the opportunity of inspecting the Milan Institute, and found that the central point of the complex of clinics is a department for obstetrics and gynæcology, which may well serve as an example for others. With the assistance of his Excellency Professor Baccelli, and under the direction of Professor Mangliagalli, a veritable model institute has here been erected, which is provided with all the details and arrangements of a modern clinic and at the same time renders inestimable services to the patients as well as to the cause of post-graduate education. Every Italian practitioner is entitled to work at this clinic, giving his services gratuitously. An opinion of its extent may be formed by the number of beds, which are 200, of which 120 are for gynæcological and 80 for obstetrical cases. The institute has a small collection of model objects, in which historical instruments and a number of pelves, both normal and pathological, are well represented. There is also a library, a chemical and bacteriological laboratory, and a room for endoscopy and *x*-ray examinations.

### Great Britain.

In regard to England, post-graduate medical classes are held in (1) London.—(*a*) At the Post-Graduate Medical College. This is an institute for the instruction of physicians and contains an auditorium, a reading-room, laboratories (for chemical, microscopical, and bacteriological examinations), and is connected with a large policlinic. Into the latter the matriculates bring their patients, thereby contributing to the available pathological material. Each matriculate has to procure a certificate of admission, costing a guinea, which entitles him to attend the lectures, including the demonstrations of patients. Should he, however, desire to become a member of the regular working clinics, he will have to pay special fees, which are rather high. (*b*) West London Post-Graduate Hospital. In this hospital there are only lectures on internal medicine, with demonstrations of patients, which any practitioner is allowed to attend. (*c*) Seaman's Hospital Post-Graduate College, at which none but paid classes are conducted. (2) Dublin.—In the Rotunda Hospital there is a post-graduate service in obstetrics, which is similar to the one established in the Milan Institute for Post-Graduate Instruction in Obstetrics and Gynæcology of Professor Mangiagalli, described before. Matriculates enter as "Volunteer Physicians" for a period of three months, for which they have to pay a fee of 30 guineas. In spite of the high fee they have also to provide their own meals. (3) At Cambridge, Edinburgh, Manchester, Leeds, and Oxford there are vacation courses in internal medicine, which are charged for.

It will be seen, therefore, that in England little has been done in the way of medical post-graduate service.

### France.

Conditions in France are similar to those in England; there is practically no organisation for graduate instruction. A few instructors, as, for instance, Professor Gaucher at the Hôpital Saint-Louis, conduct classes, and some time ago Dr. Lucas Championmère planned the establishment of an Association d'enseignement medical des hôpitaux, but, so far as I have been able to ascertain, a real organisation has not yet materialised; although a few years ago the first national convention of French physicians took this question under serious consideration, fully agreeing with a report to that effect, presented by Dr. Barbarin, who gave expression to the necessity for such an organisation.

On the other hand, Drs. Leredde and Marchais, founders of the Association d'enseignement medical professionel, have conducted in this institute vacation classes since 1902, which were attended by an average of 130 to 150 foreign practitioners—notably Russian, Spanish, American, and Canadian. In Belgium Antwerp has an organisation for medical post-graduate instruction outside the university, and lectures have been delivered there for several years past. In Liége there was a vacation course last year, and also in the present year. The latter course dealt chiefly with surgery. There is also a movement under way in favour of a medical post-graduate service which may perhaps lead to an organisation in a few years embracing the whole of Belgium.

### Holland.

In regard to post-graduate instruction in Holland I have, according to information furnished me by Sanitary Councillor Dr. Dutilh, of Rotterdam, to report the following. In Rotterdam there has been for five years a Society for Medical Post-Graduate Instruction. The membership fee is five florins. Every Tuesday from 12 to 4 o'clock, from October to June, various instructors hold classes on internal diseases, nervous affections, surgery, pathological anatomy, obstetrics, gynæcology, and dermatic affections. About 60 practitioners from various parts of Holland attend these courses as a rule. The instructors give their services free. After the working expenses have been defrayed out of the fees, the excess is used for the purchase of instruments. There is also an annual post-graduate course in Amsterdam for which a fee of 10 florins is charged. Considering the smallness of the amounts of five and 10 florins, these sums may fairly be put down as subscription fees, and the instruction itself be classed as gratuitous.

### Sweden and Norway.

There are as yet no regular arrangements in existence for post-graduate education in Sweden. However, Medicinal Councillor Dr. Wawrinsky reports that about once every two years there are courses, for which a charge is made, for the instruction of such practitioners as desire to become so-called "First Provincial Practitioners," and there are also gratuitous courses for military surgeons once every two years. The former cycle of classes comprises lectures and demonstrations in hygiene, medical chemistry and prescribing.

With reference to the medical post-graduate movement in Norway, I have received the following information from Professor Johannessen. In 1893 the Norwegian parliament (Storthing) made an appropriation of 3,000 kronen (25 cents are equal to about 0.89 Norw. kronen) for post-graduate instruction to district physicians appointed by the State. In 1904 this sum was included in the estimate. In 1904 it was reduced to one-half (1,500 kronen) and has not been raised since.

The allowance mentioned was always intended as a bonus to the district physicians for a sojourn to Christiania. In the latter place, however, the members of the faculty had been obliging enough to conduct the post-graduate instruction gratuitously in certain medical subjects. It was soon found, however, that a number of physicians other than those officially appointed availed themselves of the benefits of post-graduate instruction, and the applications for the latter were rather numerous. Both the Government and the Storthing decided in 1902 that instructors in subjects which are of special importance in the State service, such as hygiene, forensic medicine, and psychiatry, are justly entitled to a fee for their services, and in the year named 1,000 kronen were allowed for this purpose. The instructors in these subjects hold only annual classes during September and October. The classes may be attended not only by district practitioners, but also by those who hold positions as presidents in sanitary committees. Simultaneously with these classes, but commencing a little later, classes are being held for all practitioners without distinction. The subjects embrace surgery, ophthalmology, pediatry, diseases of the ear, nose, and throat, dermatic affections and gynæcology. All the lectures are held in the university clinics. The university instructors in diseases of the ear, nose, and throat, as well as the clinical assistants in surgery and gynæcology, receive 20 kronen for each matriculate, while the other university instructors give their lectures and demonstrations free. Outside Christiania similar courses have up to the present not been established.

### Summary and Conclusions.

Surveying everything that has been achieved so far, it is an occasion for rejoicing that practitioners of the various countries may pride themselves on their unselfish work in furthering the interests of their colleagues to a noteworthy extent. But still greater work remains to be done. Whoever observes the rapid development of medical science and contemplates with a feeling of awe the ever-extending specialising of medical subjects will be ready to admit that the necessity of ever supplementing his knowledge after having qualified continually accompanies the practitioners. The reproach of our century, precipitation, has seized upon medical investigation, and even more so upon medical authors. Who is able to hold his own in the flood of printed paper waves, unless the hand of the discriminating guide offers him the life-belt? The blessings of a genius, consisting in his power of concentration, are a curse upon the generality of practitioners by the ever-finer divisions of specialising, until the very organs are specialised separately. Here commences the great task of medical post-graduate education, not the task of raising specialists, but the important duty of enabling the general practitioner in the best possible manner to practise his profession by keeping in touch at regular periods with those advances in medical science which—to a certain extent at least—may be regarded as thoroughly tested and therefore safe assets of our art. If post-graduate instruction strives after this aim, it will not only confer upon the practitioner the best arsenal for keeping up his ideal and economic fight, but it will also serve the cultural aim of all nations by enabling the physician to fulfil in an ever-increasing degree of perfection his noble destiny; to act as preserver and protector of the national health. But what can we do to benefit the cause of post-graduate education? In the first place, I think we may help by forming a closer bond of union between us so that we may learn from each other. As I have pointed out, nearly each country has its own interesting arrangements for progressive education. To study these arrangements and, if possible, to emulate the same with due consideration of conditions in the various countries is already a noble aim. But we can do more yet. Consider that this progressive education is, after all, only a lifelong continuation of the work of study to which we were devoted in the happy days of our college time. All of us, I doubt not, are imbued with love and devotion for those men who have shown us the way into the holy temple of science. Such devotion, however, would degenerate into a mere personal cult if we did not possess the courage, as mature men, to cast a critical look back upon our college instruction and to give vent to those wishes which aim at a different shape of medical instruction in whatever direction it may be necessary. If post-graduate instruction is the continuation of college instruction, the latter must be the forerunner of the former; both are inseparable, and it is an unquestionable and inalienable duty of all those men who have the earnestness of post-graduate education at heart to collect all those experiences that indicate a necessity for reform in the methods of the forerunner—namely, academical instruction. Let me go a step further. These experiences can be collected only by the post-graduate instructor, because only he is in a position to decide whether there is that basis of knowledge present which he is entitled to expect and upon which he desires to continue building. If, then, these collected experiences are communicated to competent university instructors, there will surely result in many respects a reformatory co-operation of university professors with post-graduate instructors which can only redound to the advantage of general medical knowledge. I refrain from intimating at this juncture in what respects such reforms can be achieved, because the conditions in the various countries, which should govern these reforms, vary too greatly. But I may be permitted to observe that, with all due reference to rigorous science, the process of how modern medicine has resolved itself into technical details, how it is tied down by technical measures, imposes the duty upon medical instruction and post-graduate education alike to open up a much larger field to instruction and manual dexterity of the physician than has up to the present been the case. Another general point of view may perhaps also find here expression. In my opinion it is part of the task of modern post-graduate education to cultivate those branches which, like social medicine and prophylaxis, have not yet been assigned an accredited place in academical instruction. At the same time I am convinced that there never can be a conflict between university and post-graduate instruction, because by the former the physician is to obtain his original medical knowledge, and by the latter that knowledge is to be renovated and perfected in accordance with the results of progress and investigation. It may then be found that the details of progressive instruction may have to change, just as the subjects of instruction and the methods of academical instruction may change. This consideration, again, is another support for my view that both conceptions are inseparable and that for this reason there must be co-operation, inspired by a sense of their mutual usefulness.

Let this once be recognised and there must result a safe foundation for an international community of interests. Here again, in the designing of medical instruction and post-graduate education, we can learn from each other in order to profit by whatever may appear suitable and applicable to various conditions. The proposition to which I referred in the beginning of my paper is the natural conclusion of my arguments, and is probably known to a fair portion of my colleagues. It is, to create an "International Committee for Medical Post-Graduate Instruction" with a view to mutual incitement and information. The invitations issued by the German Imperial Committee in connection with the Central Committee, for the purpose of establishing an International Committee, has, we are rejoiced to say, met with a noble echo in all countries, delegates having been announced in definite or hopeful language by the Governments of all important civilised countries.

Do not let us look at what may here and there separate us, but upon what unites us. There is hardly an important medical movement that is qualified like that for post-graduate education to form a spiritual centre, around which the practitioners of all countries will do homage. Advanced medical instruction will enable the practitioners of the various nations to combat disease, to prevent epidemics and thereby to better the health of the individual and of the community, and the better they succeed in these aims the higher will be the degree of recognition and esteem of medical post-graduate instruction as one of the most important factors to promote social well-being and the civilisation of the world.

